# Distribution and determinants of glycosylated hemoglobin in adolescents ‐ Results from a nationwide population-based survey in Germany

**DOI:** 10.1371/journal.pone.0296962

**Published:** 2024-02-22

**Authors:** Eleni Patelakis, Anja Schienkiewitz, Julia Truthmann, Reinhard W. Holl, Christina Poethko-Müller, Gert B. M. Mensink, Christin Heidemann

**Affiliations:** 1 Department of Epidemiology and Health Monitoring, Robert Koch Institute, Berlin, Germany; 2 Institute for Community Medicine, University Medicine Greifswald, Greifswald, Germany; 3 Institute of Epidemiology and Medical Biometry, University Medical Centre, Ulm, Germany; National Research Centre, EGYPT

## Abstract

The role of glycosylated hemoglobin (HbA1c) in youth is largely unclear. The aims of this study are to investigate the distribution and potential determinants of HbA1c among a population-based sample of adolescents. The German Health Interview and Examination Survey for Children and Adolescents (KiGGS) Wave 2 includes a nationwide representative sample of 0-17-year-old participants. For this evaluation, data from a randomly selected subgroup aged 14–17 years and without diagnosed diabetes was included (n = 857). Percentile-based HbA1c values (measured at laboratory in whole blood samples by high performance liquid chromatography) were calculated to examine HbA1c distribution. Multivariable linear regression analyses were performed to investigate factors (age, sex, parental socioeconomic status, body mass index (BMI), birth weight, smoking, alcohol consumption, healthy food diversity, sport activity, oral contraceptive use) associated with HbA1c. The mean HbA1c level was 5.2% (minimum: 3.9%, P10: 4.8%, P50: 5.1%, P90: 5.5%, maximum: 6.7%). Overall, 2.8% of adolescents had an HbA1c value in the prediabetic range (5.7–6.4%) and 0.1% had an undiagnosed diabetes (≥6.5%). Multivariable regression analysis showed an inverse association of age with HbA1c (17 vs. 14 years: ß: -1.18; 95% CI -2.05, -0.31). Higher HbA1c values were observed for higher BMI-standard deviation scores (SDS) (ß: 0.24; 95% CI -0.04, 0.52) and smoking (ß: 0.73; 95% CI -0.12, 1.57), but these tendencies were non-significant. In sex-stratified analysis, smoking and birth weight were significantly associated with HbA1c in boys. Among adolescents without diagnosed diabetes in Germany, HbA1c values ranged from 3.9% to 6.7%. To ensure health in adulthood, the influence of determinants on HbA1c levels in younger age should be further investigated.

## Introduction

Metabolic disorders manifested in childhood and adolescence can have a considerable influence on health in adulthood. Diabetes mellitus is among the chronic metabolic disorders most frequently diagnosed in children and adolescents [1] and is associated with an increased blood glucose level. An immune-mediated type 1 diabetes, which is characterized by an absolute insulin deficiency, is considerably more frequently diagnosed in children and adolescents than type 2 diabetes, which is caused by impaired insulin secretion or a defect in insulin action [2]. Globally, the occurrence of type 1 as well as of type 2 diabetes varies considerably [3–5]. In Germany, around 31,000 children and adolescents (228.9 per 100,000 people) currently have type 1 diabetes and around 840 young people (13.5 per 100,000 people) have type 2 diabetes [6, 7].

Glycosylated hemoglobin (HbA1c) allows an evaluation of the blood glucose concentration of the last 6–8 weeks and therefore, is a suitable parameter used for assessing blood glucose control and monitoring the therapy of both type 1 and type 2 diabetes. In clinical practice and according to national and international guidelines, an HbA1c value of ≥ 6.5% in adults also leads to a diagnosis of diabetes [8, 9]. An indication of possible prediabetes exists if the HbA1c value is ≥ 5.7% and < 6.5% [8, 9]. Exact HbA1c cut offs for a diagnosis of diabetes in children and adolescents are also included in current guidelines in Germany [1]. Similarly, some internationally established diabetes associations such as the American Diabetes Association (ADA) recommend that HbA1c cut off values for adults could be used as a diagnostic criterion of diabetes also for children and adolescence [9, 10]. In epidemiological studies, the measurement of HbA1c is a valuable parameter to assess quality of care in persons with diabetes as well as to identify persons with previously unknown diabetes or prediabetes [11]. In this context, a distinct advantage of HbA1c compared to fasting glucose or 2-hour post-challenge glucose as established diagnostic criteria is that the measurement can be performed validly independent of the fasting status of the participants.

The currently existing knowledge of the distribution of HbA1c and possible determinants of HbA1c in childhood and youth is still scarce. There are only few studies on this issue, e.g. cohort or cross-sectional studies confined to selected areas or a specific birth cohort from Germany, Sweden and the Netherlands [12–14], a survey including normal-weight children from eight European countries [15], and national health surveys in the USA [16] and Korea [17]. They show a possible association between HbA1c and age [12, 13, 16, 17], gender [12, 16, 17] and obesity [12, 16, 17] as well as puberty [12] and parental history of diabetes [13, 16, 17]. However, in addition to the study design, the age groups of the participants and the investigated sets of potential determinants in the previous studies differed. Therefore, further investigation is required with regard to variables that are associated with HbA1c levels in the early stage of life.

Against this background, the aims of this study are to (1) analyse the distribution of HbA1c and (2) investigate different factors, such as sociodemographic, anthropometric and lifestyle factors, as potential determinants of HbA1c based on available data among a representative population-based sample of adolescents in Germany.

## Materials and methods

### Study design and study population

The second wave of the German Health Interview and Examination Survey for Children and Adolescents (KiGGS Wave 2) is a nationwide study conducted by the Robert Koch Institute between 2014 and 2017. KiGGS Wave 2 contains a nationwide representative cross-sectional sample of children and adolescents with permanent residence in Germany.

The sampling process involved two steps. In the first step, sample points were selected. For this, 167 sample points drawn for the KiGGS baseline survey (2003–2006) were used, which reflect Germany’s regional structure regarding federal state and type of municipality. In a second step, addresses of children and adolescents were randomly selected from the population registries of the respective municipality for each sample point. In order to obtain the same number of cases from all sample points, a different number of addresses was drawn for each age cohort, depending on the size of the municipality, the region, and the response rates achieved in the KiGGS baseline survey. For children and adolescents who are not German citizens, an oversampling factor of 1.5 was applied to compensate for the expected higher proportion of quality-neutral dropouts and the lower response rates in this population group [18].

**Fig 1:**
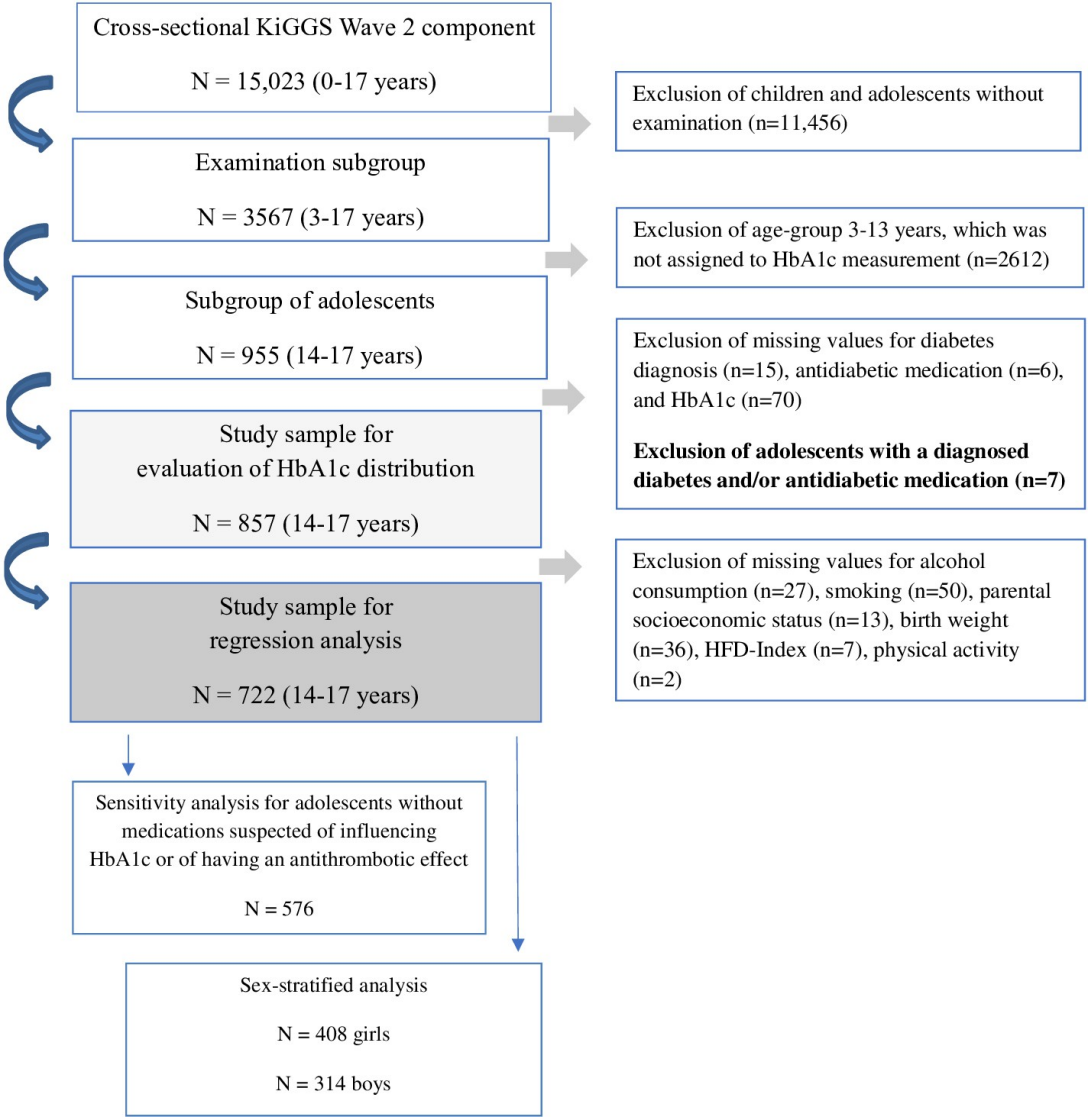
Flowchart for selection of KiGGS Wave 2 study participants aged 14–17 years.

The addresses provided from the registries were randomly divided into two groups at the Robert Koch Institute. Participants in the first group, which covered the age range from 0–17 years, were only invited to take part in the interview. Participants in the second group, aged 3–17 years, were also invited to take part in the examination. In total, 15,023 participants were interviewed (response rate: 40,1%) and of these, 3,567 participants were examined in addition to an interview (response rate: 41,5%) [18]. The examination routine varied depending on age [19]. The subgroup of 955 adolescents aged 14–17 years was assigned to HbA1c measurement in blood samples (Fig 1). Further information on study design and methods can be found elsewhere [18, 19].

All participants with a diagnosis of diabetes and/or intake of diabetes medication were excluded from the analyses (n = 7). The HbA1c distribution was calculated among 857 adolescents without missing data on diabetes diagnosis, diabetes medication, and HbA1c measurement. Regression analyses included 722 adolescents with complete information on the investigated potential determinants (age, sex, parental socioeconomic status (SES), body mass index (BMI), birth weight, smoking, alcohol consumption, healthy food diversity index (HFD), sport activity, oral contraceptive use). In a sensitivity analysis, only adolescents without an intake of medications with suspected influence on HbA1c or with having an antithrombotic effect were included (n = 576). In a second sensitivity analysis, stratification by sex was performed (girls n = 408, boys n = 314) (Fig 1).

The participants and/or their parents/legal guardians were informed about the aims and contents of the study, and about data protection. Informed consent was obtained in writing. KiGGS Wave 2 is subject to strict compliance with the data protection provisions set out in the Federal Data Protection Act. Hannover Medical School’s ethics committee examined and approved the ethics of the study (No. 2275–2014). The Federal Commissioner for Data Protection and Freedom of Information in Germany received the KiGGS Wave 2 study concept and had no objections [18].

### HbA1c measurement

At the study locations, venous blood samples were taken from the adolescents. HbA1c was analyzed in whole blood samples by high performance liquid chromatography at the laboratory of the Robert Koch Institute (HPLC-723G8 analyser, reagents from Tosoh Europe N.V., Tessenderlo, Belgium). The ranges of intra- and interassay coefficients of variations were 0.6% - 1.8% and 1.1% - 2.1%.

### Assessment of potential determinants of HbA1c

#### Body mass index

Body height and weight were measured by trained health professionals in the study centers based on standardized procedures. BMI was calculated as the ratio of body weight in kilograms and height in meters squared [20] and the values were classified according to the International Obesity Task Force (IOTF) categories [21]. For multivariable linear regression analyses, BMI was transformed into a standard deviation score (SDS) [22].

#### Smoking, alcohol intake, and sport activity

A health questionnaire was completed by the adolescents themselves. Current smoking was assessed based on the question: “*Do you currently smoke*?*”*. In addition to the answer option “*no*”, there were also “*daily*”, “*several times a week*”, “*once a week*” and “*less frequently*”, which were combined to form the "yes" category for this analysis [23]. The consumption of alcohol was evaluated by using the initial question "*Have you ever drunk alcohol*?" with the answer options "*yes*" or "*no*" [23]. Participation in sports activity was assessed by asking the initial question “*Do you do sports*?” with the answer categories “*yes*” or “*no*” [24].

#### Healthy food diversity index

The usual intake of different food groups was assessed by a food frequency questionnaire (FFQ) developed by the Robert Koch Institute, which includes 53 food items. The HFD used here was originally created for the KiGGS baseline study and calculated on the basis of 44 food items from the FFQ [25]. An adjustment of the index was made for KiGGS Wave 2. The index contains three components: the frequency, diversity and health value of all food items consumed. According to the food based dietary guidelines of the “Optimized Mixed Diet” (OMD) [26], the nutrition specific health factors have been calculated. The OMD is a concept of preventive dietary recommendations for children and adolescents. A higher HFD reflects a healthier diet.

#### Parental socioeconomic status

The parental SES was determined on the basis of their education, income and occupation. An index was formed from these three variables. The level of parental general education and the level of professional school graduation certificate were obtained with the questions: *"What is the highest level of general education you have*?*"* and *"What is the highest level of professional school graduation certificate you have*?*"*. Mother and father could choose from 6 answers for the first question and 8 answer categories for the second question. The respective highest answer of the parents was used for the index. The available financial resources were queried with the question: "*What is the total monthly net income of your household*?” with 9 possible categories [27].

#### Weight at birth

The birth weight was determined by asking "*How heavy and how tall was your child at birth*?*”*. The parents could report the approximate weight in grams and the approximate height in centimeters in the questionnaire. For this analysis, the birth weight was divided into categories (< 2500 g, 2500 g to < 4000 g, ≥ 4000 g).

### Statistical analyses

All statistical analyses were performed with SAS software Version 9.4, (SAS Institute, Cary, NC, USA) and using a weighting factor that corrects for deviations of the participant sample from the population structure with regard to age, sex, federal state, nationality and parental level of education (Microcensus 2013 [28]). Total and sex-specific HbA1c values were calculated for HbA1c percentiles (at 5-point intervals on the percentile scale, i.e. from the 5th percentile to the 95th percentile). Proportions (95% CI) for categorized variables and mean values (95% CI) for continuous variables were calculated for the descriptive characterization of the study population across four HbA1c categories that were based on aggregated HbA1c percentiles (< 10th, ≥ 10th to < 50th, ≥ 50th to < 90th and ≥ 90th percentile). Multivariable linear regression analyses were performed to investigate the association between potential determinants and HbA1c. Initial models (Model 1) were adjusted for age and sex. In an additional model (model 2) further adjustments were made. For birth weight, an additional adjustment for parental SES was performed. For all other variables, the model 2 was additionally adjusted for parental SES, lifestyle factors (smoking, HFD index, sport activity, alcohol consumption) and BMI. The selection of variables for mutual adjustments was based on the variables used in other studies on determinants of HbA1c in non-adults [12, 13, 17] and the availabilities of variables in KiGGS Wave 2. The outcome variable HbA1c was modeled as continuous variable in mmol/mol for the main analysis and logarithmically transformed for a sensitivity analysis. In addition, two further sensitivity analyses were performed. In the one analysis, subjects with medications suspected of influencing HbA1c or of having an antithrombotic effect were excluded. In the other analysis, sex-stratified results were calculated, whereas for girls, additional adjustment was also made for oral contraceptive use (additional model 3). A statistically significant difference between groups was assumed based on two-sided p-values of less than 0.05.

## Results

**Fig 2:**
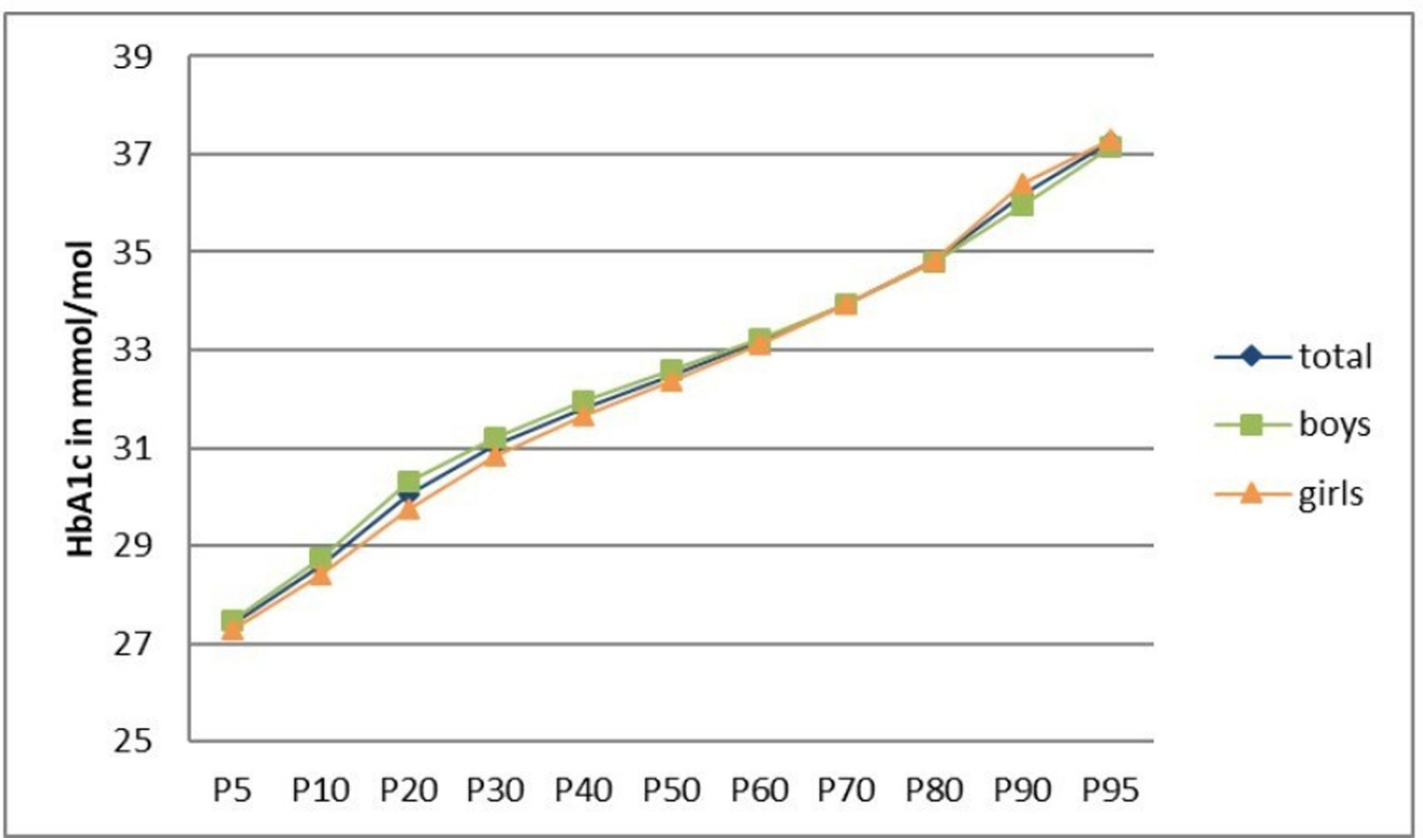
Percentiles for HbA1c from KiGGS Wave 2 study participants aged 14–17 years without diagnosed diabetes, total and by sex.

For the 857 study participants aged 14–17 years and without diagnosed diabetes, the mean HbA1c value was 5.2% (32.9 mmol/mol) (range 3.9–6.7% / 19.0–50.0 mmol/mol). The values for the 5th, 10th, 50th, 90th, and 95th percentiles were 4.7% (27.4 mmol/mol), 4.8% (28.6 mmol/mol), 5.1% (32.5 mmol/mol), 5.5% (36.2 mmol/mol), and 5.6% (37.2 mmol/mol) (Fig 2). Overall, 2.8% of adolescents (n = 24) had an HbA1c value in the pre-diabetic range (5.7–6.4%) and 0.1% of adolescents (n = 1) had an unknown diabetes (≥6.5%) according to ADA criteria.

**Fig 3:**
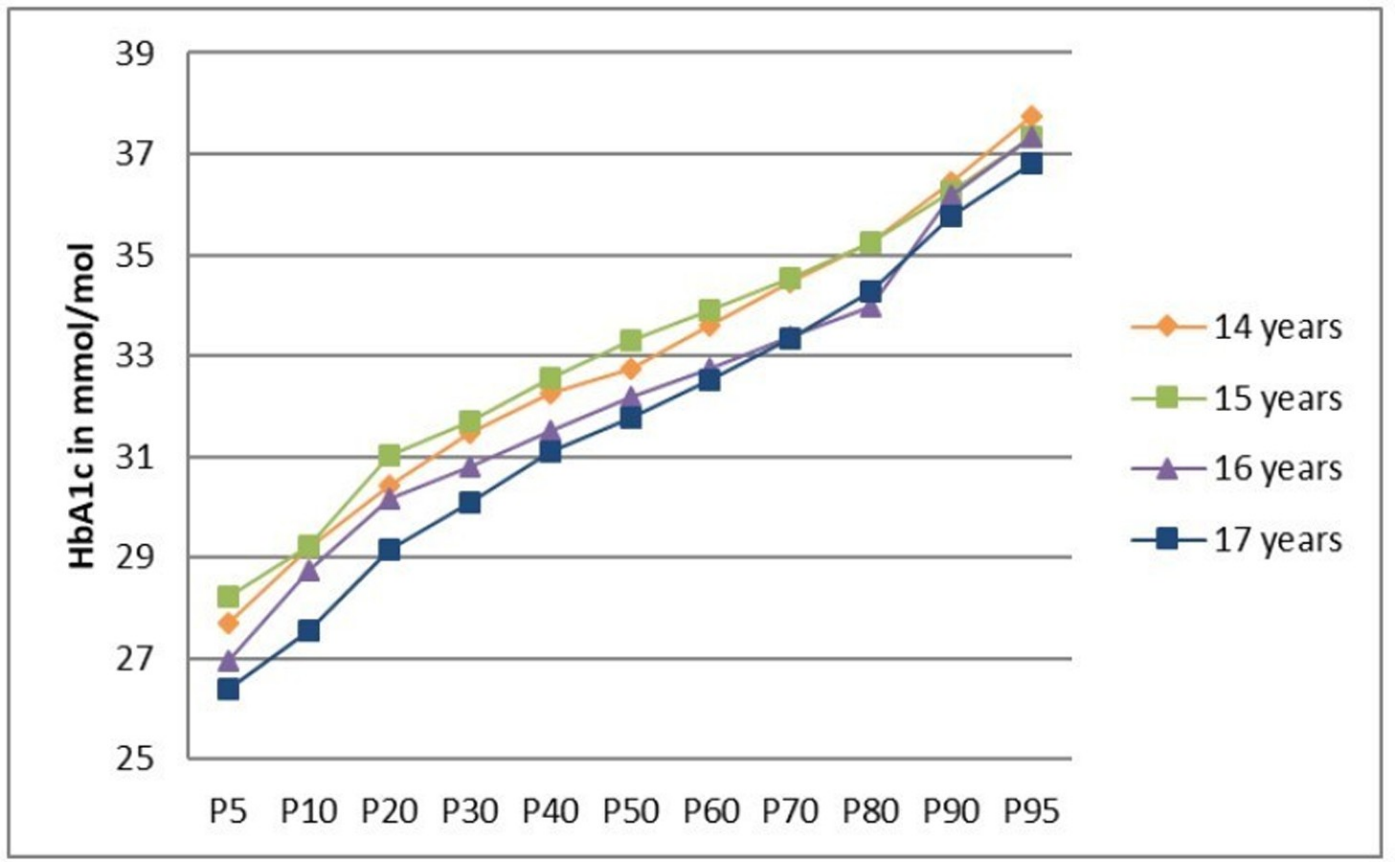
Percentiles for HbA1c from KiGGS Wave 2 study participants aged 14–17 years without diagnosed diabetes, total and by age.

Percentile curves did not differ according to sex (Fig 2). Age-specific percentile curves (Fig 3) indicated lower HbA1c values for the upper age groups, with a stronger differentiation of the HbA1c values in the lower percentile range.

Across aggregated categories of increasing HbA1c values, boys and girls were accordingly relatively evenly distributed (Table 1). The percentage of adolescents aged 14 years and 15 years increased (from 18.5% and 20.1% in the HbA1c category < 10th percentile to 27.1% and 27.2% in the HbA1c category ≥ 90th percentile), while the percentage of 16-year-olds showed no clear trend. The percentage of 17-year-olds decreased (from 37.2% to 20.6%). Accordingly, mean age slightly decreased with increasing HbA1c values (from 15.8 years to 15.4 years). The proportion of adolescents with a low parental SES was lowest and of adolescents with a medium parental SES was highest in the upper HbA1c category, while no clear trend was evident for those with a high parental SES.

**Table 1:** Characteristics of KiGGS Wave 2 study participants aged 14–17 years without diagnosed diabetes by HbA1c categories (n = 722).

	<P 10 (HbA1c < 29 mmol/mol [4.8%])			≥ P10 to < P50 (HbA1c ≥ 29 mmol/mol [4.8%] to < 33 mmol/mol [5.2%])			≥ P50 to < P90 (HbA1c ≥ 33 mmol/mol [5.2%] to < 37 mmol/mol [5.5%])			≥ P90 (HbA1c ≥ 37 mmol/mol [5.5%])		
**Total (n = 722)**	67			332			247			76		
	% or mean	*95% CI*		% or mean	*95% CI*		% or mean	*95% CI*		*%* or mean	*95% CI*	
Girls (%)	53.6	38.4	68.9	52.2	45.3	59.2	46.1	39.9	52.3	55.6	41.8	69.4
**Age category** (%)												
14 years	18.5	5.7	31.3	21.8	15.7	27.9	26.3	19.4	33.3	27.1	13.4	40.7
15 years	20.1	9.7	30.5	20.3	15.5	25.1	31.9	24.8	38.9	27.2	13.7	40.8
16 years	24.2	8.4	40.1	29.1	22.5	35.7	20.8	14.0	27.6	25.0	12.3	37.8
17 years	37.2	20.5	53.9	28.8	22.6	35.0	21.0	14.5	27.4	20.6	8.7	32.6
**Age** (years, mean)	15.8	15.5	16.1	15.6	15.5	15.8	15.4	15.2	15.5	15.4	15.1	15.7
**Parental socioeconomic status (%)**												
Low	30.1	13.5	46.6	23.3	16.5	30.1	19.7	11.8	27.7	18.9	5.4	32.5
Medium	62.1	46.4	77.7	59.1	52.2	66.1	59.3	50.9	67.7	73.7	59.7	87.7
High	7.9	1.3	14.4	17.5	13.0	22.1	21.0	14.9	27.1	7.4	1.3	13.4
**Birth weight category** (%)												
< 2500 g	7.2	1.0	13.4	5.8	1.5	10.0	3.3	0.5	6.1	8.4	0.7	16.0
2500 to < 4000 g	81.9	72.1	91.7	83.1	77.3	88.9	82.1	75.8	88.3	72.3	56.9	87.8
≥ 4000 g	10.9	2.1	19.7	11.1	7.0	15.3	14.6	8.6	20.7	19.3	4.6	34.1
**BMI** (SDS-score, mean)	-0.3	-0.5	0.05	-0.1	-0.2	0.1	0.05	-0.2	0.1	0.3	0.03	0.5
**BMI category, according to IOTF (%)**												
Underweight	14.5	0	29.4	10.3	4.8	15.9	8.1	4.4	11.8	6.6	0.7	12.6
Normal weight	74.4	59.3	89.5	66.0	58.6	73.4	73.2	66.3	80.0	63.6	49.0	78.2
Overweight/Obesity	11.0	3.4	18.7	23.7	17.2	30.1	18.7	12.4	25.0	29.8	15.3	44.2
**Smoking** (yes vs. no, %)	5.1	0	10.2	13.5	8.6	18.4	8.6	4.8	12.4	18.4	7.2	29.5
**HFD-Index** (mean)	0.5	0.5	0.5	0.5	0.5	0.5	0.5	0.5	0.5	0.5	0.4	0.5
**Sport activity** (yes vs. no, %)	74.1	58.0	90.1	70.4	63.4	77.4	76.9	69.9	83.9	70.9	56.2	85.6
**Alcohol consumption (**yes vs. no, %)	78.3	65.2	91.5	78.4	72.2	84.5	76.9	69.9	83.9	76.5	64.3	88.8
**Oral contraceptives (**yes vs. no among girls, %)	13.1	3.3	22.8	12.2	8.0	16.3	6.9	3.1	10.8	7.0	1.3	12.7

Further, the proportions of overweight or obese adolescents and of those with a birth weight of at least 4000 g in the lowest HbA1c category were considerably smaller (11.0% and 10.9% in the HbA1c category < 10th percentile) compared to the highest category (29.8% and 19.3% in the HbA1c category ≥ 90th percentile). The mean BMI-SDS increased with increasing HbA1c values (from -0.3 to 0.3). Similarly, the proportion of smokers in the lowest HbA1c category was lower (5.1%) than in the highest category (18.4%). The proportion of girls who used oral contraceptives was lower in the two upper HbA1c categories compared to the other HbA1c categories. For sport activity, no clear trend was evident and for the proportion of alcohol consumers and the mean HFD-index, results were similar across the HbA1c percentile categories (Table 1).

Table 2 shows the associations between potential determinants and HbA1c from linear regression models among the 722 adolescents with complete data. Model 1 was adjusted for age and sex and revealed significant lower HbA1c values for adolescents aged 17 years compared to those aged 14 years (ß: -1.20; p: 0.004). For smokers, higher HbA1c values were found compared to never smokers (ß: 0.82; p: 0.055) and for BMI, higher SDS values were observed with increasing HbA1c values (β: 0.22, p: 0.12), but these associations were statistically not significant. In model 2, which was further adjusted for parental SES, lifestyle factors, and BMI-SDS (except for birth weight, which was further adjusted for SES only), the associations with HbA1c for the age of 17 years (ß: -1.18, p: 0.008) and for smoking (ß: 0.73, p: 0.094) were slightly weaker, whereas the tendency for BMI-SDS (ß: 0.24, p: 0.090) was slightly more pronounced. For sex, parental SES, birth weight, the HFD index, sport activity, and alcohol consumption, no associations were found. In the sensitivity analysis with logarithmically transformed HbA1c values, associations were comparable (S1 Table).

**Table 2:** Associations between sociodemographic, anthropometric and lifestyle parameters and HbA1c among KiGGS Wave 2 study participants aged 14–17 years without diagnosed diabetes (n = 722).

	Model 1				Model 2			
	β	95% CI		p-value	β	95% CI		p-value
**Sex**								
Boys	reference				reference			
Girls	-0.25	-0.75	0.26	0.34	-0.17	-0.68	0.34	0.50
**Age (years)**								
14	reference				reference			
15	0.05	-0.83	0.73	0.91	0.01	-0.76	0.79	0.97
16	-0.76	-1.58	0.06	0.069	-0.62	-1.45	0.22	0.15
17	-1.20	-2.01	-0.40	0.004	-1.18	-2.05	-0.31	0.008
**Parental socioeconomic status**								
Low	reference				reference			
Medium	0.51	-0.09	1.10	0.096	0.52	-0.33	1.37	0.23
High	0.49	-0.20	1.17	0.16	0.85	-0.10	1.81	0.079
**Birth weight (g)**								
< 2500	-0.06	-1.14	1.03	0.92	-0.06	-1.14	1.01	0.91
2500 to < 4000	reference				reference			
≥ 4000	0.65	-0.24	1.55	0.15	0.62	-0.29	1.54	0.18
**Body mass index**								
BMI-SDS	0.22	-0.06	0.49	0.12	0.24	-0.04	0.52	0.090
**Smoking**								
No	reference				reference			
Yes	0.82	-0.02	1.66	0.055	0.73	-0.12	1.57	0.094
**Diet**								
HFD Index	-1.10	-3.16	0.95	0.29	-1.22	-3.22	0.80	0.24
**Sport activity**								
No	reference				reference			
Yes	0.02	-0.67	0.72	0.94	0.03	-0.68	0.74	0.93
**Alcohol consumption**								
No	reference				reference			
Yes	0.01	-0.69	0.72	0.98	-0.19	-0.94	0.56	0.61

HbA1c was included as a continuous variable (mmol/mol) in the regression model.

Model 1 was adjusted for age and sex. For birth weight: model 2 was additionally to model 1 adjusted for parental SES. For all variables except birth weight: model 2 was additionally to model 1 adjusted for parental SES, lifestyle factors (smoking, HFD index, sport activity, alcohol consumption) and BMI. Estimates for age, sex and parental SES shown in model 2 are based on the latter comprehensively adjusted model.

In the sensitivity analysis excluding adolescents with medications suspected of influencing HbA1c or of having an antithrombotic effect, results remained similar (S2 Table). In model 2, in addition to higher HbA1c values for adolescents aged 17 years (ß: -1.18, p: 0.012), also significantly higher HbA1c values for adolescents aged 16 years (ß: -0,95, p: 0.032) compared to those aged 14 years were observed.

In the sex-stratified analysis, similar coefficients in both sexes were observed for the age-group 17 years (girls: ß: -1.24, p: 0.036, boys: ß: -1.04, p: 0.13 in model 2) compared to the main analysis, although the statistical significance was lower. In the analysis for girls, oral contraceptive use was included in addition to the other potential determinants (model 3). The use of the contraceptives had a negative, but non-significant coefficient (ß: -0.81, p: 0.099) and its inclusion lowered the coefficient for the age-group 17 years (ß: -1.04, p: 0.81; S3 Table). In the analysis for boys, results gained additional statistical significance for birth weight ≥ 4000 g (ß: 1.27, p: 0.038 in model 2) and smoking (ß: 1.48, p: 0.010 in model 2; S4 Table).

## Discussion

This study is based on data from a nationwide population-based sample of adolescents aged 14–17 years from Germany. Among adolescents without diagnosed diabetes, HbA1c values ranged from 3.9% to 6.7% and the mean level of HbA1c was 5.2%. The observed difference of HbA1c between 14- and 17-year old adolescents remained statistically significant after the control for the further considered sociodemographic, lifestyle and anthropometric variables. For higher BMI and smoking, higher HbA1c values were found, but these tendencies were non-significant in the overall study population. However, sex-stratified analysis showed a significant association of HbA1c with smoking as well as with birth weight among boys. In girls, the significant association of HbA1c with age was attenuated when additionally adjusting for oral contraceptive use.

The mean HbA1c and the distribution of the measured values are similar to the results of other studies [13, 14, 29]. For example, the distribution of HbA1c levels for children and adolescents with an age range of 1–17 years in KiGGS (2003–2006) was between 3.6% (3rd percentile) and 5.8% (97th percentile) [29]. A study from Sweden investigated the distribution of HbA1c in apparently healthy children aged between 6 months and 18 years. The range for HbA1c over the whole age group was 3.7% to 4.7% [14]. The distribution of HbA1c levels was wider and ranged from 3.4% to 8.5% in a study with 14-18-year-old California high school students; however, two undiagnosed type 2 diabetics were included [30].

The HbA1c values were similar in the age groups 14 years and 15 years and then decreased with increasing age in this study population. The significant difference in 17-year-olds compared to 14-year-olds was largely preserved in the sensitivity analyses. However, in the sex-stratified multivariable regression analysis, the association with age was no longer significant for boys and also not significant for girls after adjustment for oral contraceptive use, but this could be due to the respective smaller sample sizes. Current data from other studies indicate that the HbA1c level is subject to large variation in the age spectrum of childhood and adolescence. In the KiGGS baseline study, the highest HbA1c values were found for the age group 10–14 years [29]. This age group had also the highest measured levels in the third National Health and Nutrition Examination Survey (NHANES III) in the U.S. from 1988–1994 [16]. A multicentric study from Italy showed no difference in HbA1c values for the same gender according to three age groups (3–9 years, 10–14 years, 15–17 years) [31]. A positive significant relationship between age and HbA1c in children and adolescents (0,5 to 17 years) was found in the local population-based LIFE CHILD study conducted in the city of Leipzig, Germany (2011–2017) [12].

HbA1c levels showed no significant differences between female and male subjects in this study. In some studies, a sex-difference in HbA1c values is reported, sometimes only for individual age groups. Boys had slightly higher values than girls in KiGGS [29]. Higher HbA1c levels were associated with male gender in the LIFE CHILD study [12]. A Korean Health and Nutrition Examination Survey (KNHANES) (2011–2015) of subjects aged 10–29 years also showed higher values for male subjects [17]. In Californian high school graduates aged 14–18 years, slightly but significantly higher HbA1c values were found in male compared to female subjects [30]. However, higher values for boys than girls were found only in the age group 10–14 years in a clinical study from Italy [31].

We found no association between parental SES and HbA1c values in our study. There is also indication from other studies that parental education and income have no association with HbA1c values in children and adolescents [13, 17]. Interestingly, a positive correlation between parental SES and HbA1c was found in the local study (Leipzig) in Germany [12], although the authors of the study expected a negative correlation. Altogether, data on HbA1c and SES in youth are limited. This gap should be closed with further research on this issue.

In the present study, a tendency for an association of higher BMI-SDS with higher values of HbA1c was observed, however, this association was not statistically significant. Other studies with a similar age range indicate that very high BMI values are associated with higher HbA1c values [30, 32]. A correlation between the BMI-SDS and HbA1c level over the whole child and adolescent age is shown in the LIFE CHILD study [12]. Overweight, defined as the age- and sex-specific BMI exceeding the 95th percentile was also associated with a higher HbA1c in the NHANES III [16]. A potential explanation why only a non-significant tendency for BMI was observed for this study sample could be the moderate number of subjects included in the calculations.

An association between birth weight and HbA1c level could not be found in the overall sample of this study; but interestingly, a birth weight of at least 4000 g was significantly related to higher values of HbA1c compared to a birth weight between 2500 g and 4000 g among boys in the sex-stratified analysis. There are relatively few previous studies on the relationship between birth weight and HbA1c. There was also no impact of birth weight on HbA1c levels in the LIFE CHILD study among the overall study population of children and adolescents [12]. Possibly, HbA1c levels in adolescents are more dependent on further factors such as the hormone status, which changes differently in the two sexes, than on birth weight more than a decade ago, but this needs further elucidation.

The lifestyle factors alcohol consumption, diet (HFD index) and sport activity showed no association with HbA1c in the present study. Maybe it is too early in life for these factors to show an association with HbA1c. The calculated lifestyle scores “healthy diet”, “saturated fat” and “physical activity” were also not associated with HbA1c values in the Dutch study with children aged 12 years [13]. Similarly, no association between physical activity and HbA1c was found in the LIFE CHILD study [12].

Higher HbA1c values for current smokers than for nonsmokers were found in our entire study population based on the age- and sex-adjusted analysis. However, the association was somewhat weaker and also not statistically significant in the fully adjusted analysis. After sex-stratification, a significant positive association between smoking and HbA1c level was found for male subjects. We could not find a comparable study that has investigated smoking as a determinant of HbA1c in adolescents. However, HbA1c was higher in smokers compared with non-smokers in adults [33, 34]. More data are also needed in adolescence.

Although the negative coefficient of oral contraceptive use in girls indicated that their use may be associated with lower HbA1c levels, the association showed no statistical significance in the present study. Unfortunately, no data on the state of puberty were available for the sample of our study. The influence of puberty or the use of sex hormones in healthy adolescents on HbA1c has only been marginally analyzed in the past. Previous study results suggest that during pubertal development there may be variations in HbA1c values and the beginning of puberty may be associated with changes in insulin sensitivity [35]. In the LIFE CHILD study, the influence of pubertal status based on tanner stages 1 to 5 on HbA1c was investigated separately by sex. In male subjects, an increase in HbA1c with advancing pubertal status was observed. In girls, an increase in HbA1c was also found during pubertal development (except for tanner stage 4) [12]. Data from the Health Influences of Puberty (HIP) Study showed that HbA1c increased as puberty progresses [36].

The strengths of this study include the nationwide representative data and the comprehensive set of sociodemographic, anthropometric, and lifestyle variables. In addition, because of the specific age-range of 14–17 years, a targeted focus could be made for adolescents. However, there are also limitations. Based on the cross-sectional design, no causal interference can be derived between possible determinants and HbA1c. The general questions on alcohol consumption and sport activity used here may not have provided the differentiation needed to observe differences in HbA1c levels. The number of included participants was modestly, which affected the resulting statistical power. Important possible determinants, such as pubertal status and diabetes history of parents were not available, but would probably have made an important contribution in clarifying associations.

## Conclusions

In conclusion, these findings suggest that more research is needed to understand associations between determinants and HbA1c level in adolescence. Potentially modifiable factors that lead to higher HbA1c levels in childhood and adolescence, such as higher BMI, should be monitored early so that health measures at the individual and public health level can be strengthened, thereby preventing potential glucose metabolism disorders in adulthood.

## Supporting information

S1 Table Associations between sociodemographic, anthropometric and lifestyle parameters and HbA1c (logarithmically transformed values) among KiGGS Wave 2 study participants aged 14–17 years without diagnosed diabetes (n = 722). HbA1c was included in the regression model in logarithmically transformed mmol/mol-values. Model 1 was adjusted for age and sex. For birth weight: model 2 was additionally to model 1 adjusted for parental SES. For all variables except birth weight: model 2 was additionally to model 1 adjusted for parental SES, lifestyle factors (smoking, HFD index, sport activity, alcohol consumption) and BMI. Estimates for age, sex and parental SES shown in model 2 are based on the latter comprehensively adjusted model.
(DOCX)

S2 Table Sensitivity analysis for adolescents without medications suspected of influencing HbA1c or of having an antithrombotic effect (n = 576). HbA1c was included as a continuous variable (mmol/mol) in the regression model. Model 1 was adjusted for age and sex. For birth weight: model 2 was additionally to model 1 adjusted for parental SES. For all variables except birth weight: model 2 was additionally to model 1 adjusted for parental SES, lifestyle factors (smoking, HFD index, sport activity, alcohol consumption) and BMI. Estimates for age, sex and parental SES shown in model 2 are based on the latter comprehensively adjusted model.
(DOCX)

S3 Table Stratified analysis for girls (n = 408). HbA1c was included as a continuous variable (mmol/mol) in the regression model. Model 1 was adjusted for age. For birth weight: model 2 was additionally to model 1 adjusted for parental SES. For all variables except birth weight: model 2 was additionally to model 1 adjusted for parental SES, lifestyle factors (smoking, HFD index, sport activity, alcohol consumption) and BMI. Estimates for age and parental SES shown in model 2 are based on the latter comprehensively adjusted model. For all variables except birth weight: model 3 was additionally to model 2 adjusted for oral contraceptive use.
(DOCX)

S4 Table Stratified analysis for boys (n = 314). HbA1c was included as a continuous variable (mmol/mol) in the regression model. Model 1 was adjusted for age. For birth weight: model 2 was additionally to model 1 adjusted for parental SES. For all variables except birth weight: model 2 was additionally to model 1 adjusted for parental SES, lifestyle factors (smoking, HFD index, sport activity, alcohol consumption) and BMI. Estimates for age and parental SES shown in model 2 are based on the latter comprehensively adjusted model.
(DOCX)
