# Supplementary material for: Distribution and determinants of glycosylated hemoglobin in adolescents ‐ Results from a nationwide population-based survey in Germany
Source: PLoS One. 2024 Feb 22;19(2):e0296962. doi: 10.1371/journal.pone.0296962 (PMC10883580; doi:10.1371/journal.pone.0296962)
Supplement: S1 Table — HbA1c was included in the regression model in logarithmically transformed mmol/mol-values. Model 1 was adjusted for age and sex. For birth weight: model 2 was additionally to model 1 adjusted for parental SES. For all variables except birth weight: model 2 was additionally to model 1 adjusted for parental SES, lifestyle factors (smoking, HFD index, sport activity, alcohol consumption) and BMI. Estimates for age, sex and parental SES shown in model 2 are based on the latter comprehensively adjusted model. (DOCX) [file pone.0296962.s001.docx]

|  | **Model 1** | | | | **Model 2** | | | |
| --- | --- | --- | --- | --- | --- | --- | --- | --- |
|  | **β** | **95% CI** | | **p-value** | **β** | **95% CI** | | **p-value** |
| **Sex** |  |  |  |  |  |  |  |  |
| Boys | reference |  |  |  | reference |  |  |  |
| Girls | -0.005 | -0.014 | 0.004 | 0.31 | -0.003 | -0.012 | 0.006 | 0.47 |
| **Age (years)** |  |  |  |  |  |  |  |  |
| 14 | reference |  |  |  | reference |  |  |  |
| 15 | -0.001 | -0.014 | 0.013 | 0.92 | 0.000 | -0.013 | 0.014 | 0.96 |
| 16 | -0.013 | -0.028 | 0.001 | 0.07 | -0.011 | -0.026 | 0.004 | 0.15 |
| 17 | -0.022 | -0.036 | -0.007 | 0.004 | -0.021 | -0.037 | -0.006 | 0.008 |
| **Parental socioeconomic status** |  |  |  |  |  |  |  |  |
| Low | reference |  |  |  | reference |  |  |  |
| Medium | 0.009 | -0.003 | 0.021 | 0.10 | 0.009 | -0.006 | 0.025 | 0.23 |
| High | 0.009 | -0.002 | 0.019 | 0.15 | 0.016 | -0.001 | 0.033 | 0.07 |
| **Birth weight** **(g)** |  |  |  |  |  |  |  |  |
| < 2500 | 0,001 | -0.021 | 0.018 | 0.89 | -0.001 | -0.020 | 0.018 | 0.89 |
| 2500 to < 4000 | reference |  |  |  | reference |  |  |  |
| ≥ 4000 | 0.011 | -0.004 | 0.027 | 0.16 | 0.011 | -0.005 | 0.027 | 0.19 |
| **Body mass index** |  |  |  |  |  |  |  |  |
| BMI-SDS | 0.004 | -0.001 | 0.009 | 0.13 | 0.004 | -0.001 | 0.009 | 0.09 |
| **Smoking** |  |  |  |  |  |  |  |  |
| No | reference |  |  |  | reference |  |  |  |
| Yes | 0.014 | 0.000 | 0.029 | 0.054 | 0.013 | -0.002 | 0.028 | 0.09 |
| **Diet** |  |  |  |  |  |  |  |  |
| HFD Index | -0.019 | -0.055 | 0.017 | 0.31 | -0.021 | -0.056 | 0.015 | 0.25 |
| **Sport activity** |  |  |  |  |  |  |  |  |
| No | reference |  |  |  | reference |  |  |  |
| Yes | 0.000 | -0.012 | 0.013 | 0.97 | 0.000 | -0.012 | 0.013 | 0.96 |
| **Alcohol consumption** |  |  |  |  |  |  |  |  |
| No | reference |  |  |  | reference |  |  |  |
| Yes | 0.000 | -0.012 | 0.012 | 0.99 | -0.004 | -0.017 | 0.010 | 0.60 |

**S1 Table.** **Associations between sociodemographic, anthropometric and lifestyle parameters and HbA1c (logarithmically transformed values) among KiGGS Wave 2 study participants aged 14-17 years without diagnosed diabetes (n=722).** HbA1c was included in the regression model in logarithmically transformed mmol/mol-values. Model 1 was adjusted for age and sex. For birth weight: model 2 was additionally to model 1 adjusted for parental SES. For all variables except birth weight: model 2 was additionally to model 1 adjusted for parental SES, lifestyle factors (smoking, HFD index, sport activity, alcohol consumption) and BMI. Estimates for age, sex and parental SES shown in model 2 are based on the latter comprehensively adjusted model.
