# Supplementary material for: Distribution and determinants of glycosylated hemoglobin in adolescents ‐ Results from a nationwide population-based survey in Germany
Source: PLoS One. 2024 Feb 22;19(2):e0296962. doi: 10.1371/journal.pone.0296962 (PMC10883580; doi:10.1371/journal.pone.0296962)
Supplement: S2 Table — HbA1c was included as a continuous variable (mmol/mol) in the regression model. Model 1 was adjusted for age and sex. For birth weight: model 2 was additionally to model 1 adjusted for parental SES. For all variables except birth weight: model 2 was additionally to model 1 adjusted for parental SES, lifestyle factors (smoking, HFD index, sport activity, alcohol consumption) and BMI. Estimates for age, sex and parental SES shown in model 2 are based on the latter comprehensively adjusted model. (DOCX) [file pone.0296962.s002.docx]

|  | **Model 1** | | | | **Model 2** | | | |
| --- | --- | --- | --- | --- | --- | --- | --- | --- |
|  | **β** | **95% CI** | | **p-value** | **β** | **95% CI** | | **p-value** |
| **Sex** |  |  |  |  |  |  |  |  |
| Boys | reference |  |  |  | reference |  |  |  |
| Girls | -0.47 | -1.00 | 0.06 | 0.082 | -0.38 | -0.90 | 0.13 | 0.15 |
| **Age (years)** |  |  |  |  |  |  |  |  |
| 14 | reference |  |  |  | reference |  |  |  |
| 15 | -0.16 | -0.98 | 0.67 | 0.71 | -0.13 | -0.96 | 0.70 | 0.76 |
| 16 | -1.05 | -1.91 | -0.19 | 0.017 | -0.95 | -1.82 | -0.08 | 0.032 |
| 17 | -1.09 | -1.93 | -0.26 | 0.011 | -1.18 | -2.09 | -0.26 | 0.012 |
| **Parental socioeconomic status** |  |  |  |  | . |  |  |  |
| Low | reference |  |  |  | reference |  |  |  |
| Medium | 0.36 | -0.48 | 1.20 | 0.40 | 0.35 | -0.56 | 1.27 | 0.45 |
| High | 0.48 | -0.49 | 1.46 | 0.33 | 0.54 | -0.48 | 1.55 | 0.30 |
| **Birth weight** **(g)** |  |  |  |  |  |  |  |  |
| < 2500 | -0.87 | -2.21 | 0.48 | 0.20 | -0.91 | -2.23 | 0.42 | 0.18 |
| 2500 to < 4000 | reference |  |  |  | reference |  |  |  |
| ≥ 4000 | 0.28 | -0.61 | 1.16 | 0.54 | 0.25 | -0.63 | 1.14 | 0.57 |
| **Body mass index** |  |  |  |  |  |  |  |  |
| BMI – SDS | 0.20 | -0.05 | 0.45 | 0.12 | 0.23 | -0.03 | 0.49 | 0.086 |
| **Smoking** |  |  |  |  |  |  |  |  |
| No | reference |  |  |  | reference |  |  |  |
| Yes | 0.63 | -0.19 | 1.45 | 0.13 | 0.56 | -0.27 | 1.40 | 0.18 |
| **Diet** |  |  |  |  |  |  |  |  |
| HFD Index | -0.68 | -2.73 | 1.36 | 0.51 | -0.77 | -2.81 | 1.28 | 0.46 |
| **Sport activity** |  |  |  |  |  |  |  |  |
| No | reference |  |  |  | reference |  |  |  |
| Yes | 0.36 | -0.39 | 1.12 | 0.34 | 0.34 | -0.47 | 1.14 | 0.41 |
| **Alcohol consumption** |  |  |  |  |  |  |  |  |
| No | reference |  |  |  | reference |  |  |  |
| Yes | 0.18 | -0.58 | 0.93 | 0.64 | 0.00 | -0.79 | 0.79 | 1.00 |

**S2 Table. Sensitivity analysis for adolescents without medications suspected of influencing HbA1c or of having an antithrombotic effect (n=576).** HbA1c was included as a continuous variable (mmol/mol) in the regression model. Model 1 was adjusted for age and sex. For birth weight: model 2 was additionally to model 1 adjusted for parental SES. For all variables except birth weight: model 2 was additionally to model 1 adjusted for parental SES, lifestyle factors (smoking, HFD index, sport activity, alcohol consumption) and BMI. Estimates for age, sex and parental SES shown in model 2 are based on the latter comprehensively adjusted model.
