# Supplementary material for: Distribution and determinants of glycosylated hemoglobin in adolescents ‐ Results from a nationwide population-based survey in Germany
Source: PLoS One. 2024 Feb 22;19(2):e0296962. doi: 10.1371/journal.pone.0296962 (PMC10883580; doi:10.1371/journal.pone.0296962)
Supplement: S3 Table — HbA1c was included as a continuous variable (mmol/mol) in the regression model. Model 1 was adjusted for age. For birth weight: model 2 was additionally to model 1 adjusted for parental SES. For all variables except birth weight: model 2 was additionally to model 1 adjusted for parental SES, lifestyle factors (smoking, HFD index, sport activity, alcohol consumption) and BMI. Estimates for age and parental SES shown in model 2 are based on the latter comprehensively adjusted model. For all variables except birth weight: model 3 was additionally to model 2 adjusted for oral contraceptive use. (DOCX) [file pone.0296962.s003.docx]

|  | **Model 1** | | | | **Model 2** | | | | **Model 3** | | | | |
| --- | --- | --- | --- | --- | --- | --- | --- | --- | --- | --- | --- | --- | --- |
|  | **β** | **95% CI** | | **p-value** | **β** | **95% CI** | | **p-value** | **β** | **95% CI** | | **p-value** | |
| **Age (years)** |  |  |  |  |  |  |  |  |  |  |  |  | |
| 14 | reference |  |  |  | reference |  |  |  | reference |  |  |  | |
| 15 | -0.11 | -1.15 | 0.93 | 0.83 | -0.15 | -1.12 | 0.81 | 0.76 | 0.04 | -1.03 | 0.94 | 0.93 | |
| 16 | -0.85 | -1.90 | 0.19 | 0.11 | -0.96 | -1.96 | 0.03 | 0.058 | -0.83 | -1.85 | 0.18 | 0.11 | |
| 17 | -1.15 | -2.22 | -0.08 | 0.03 | -1.24 | -2.39 | -0.08 | 0.036 | -1.04 | -2.22 | 0.13 | 0.081 | |
| **Parental socioeconomic status** |  |  |  |  |  |  |  |  |  |  |  |  | |
| Low | reference |  |  |  | reference |  |  |  | reference |  |  |  | |
| Medium | 0.15 | -0.88 | 1.18 | 0.77 | 0.13 | -0.92 | 1.19 | 0.80 | 0.10 | -0.93 | 1.13 | 0.085 | |
| High | 0.61 | -0.48 | 1.70 | 0.27 | 0.63 | -0.52 | 1.78 | 0.28 | 0.55 | -0.61 | 1.71 | 0.35 | |
| **Birth weight** **(g)** |  |  |  |  |  |  |  |  |  |  |  |  | |
| < 2500 | -1.06 | -2.64 | 0.53 | 0.19 | -1.00 | -2.61 | 0.62 | 0.22 |  | | | | |
| 2500 to < 4000 | reference |  |  |  | reference |  |  |  | n.a | | | | |
| ≥ 4000 | -0.09 | -1.69 | 1.50 | 0.91 | -0.13 | -1.78 | 1.52 | 0.88 |  | | | | |
| **Body mass index** |  |  |  |  |  |  |  |  |  |  |  |  |  |
| BMI**-**SDS | 0.13 | -0.18 | 0.45 | 0.40 | 0.16 | -0.17 | 0.49 | 0.34 | 0.15 | -0.17 | 0.48 | 0.36 |  |
| **Smoking** |  |  |  |  |  |  |  |  |  |  |  |  |  |
| No | reference |  |  |  | reference |  |  |  | reference |  |  |  | |
| Yes | 0.11 | -0.99 | 1.20 | 0.85 | 0.00 | -1.14 | 1.14 | 1.00 | 0.13 | -1.06 | 1.33 | 0.82 | |
| **Diet** |  |  |  |  |  |  |  |  |  |  |  |  | |
| HFD Index | -0.23 | -2.81 | 2.35 | 0.86 | -0.36 | -2.92 | 2.21 | 0.78 | -0.50 | -3.03 | 2.03 | 0.70 | |
| **Sport activity** |  |  |  |  |  |  |  |  |  |  |  |  | |
| No | reference |  |  |  | reference |  |  |  | reference |  |  |  | |
| Yes | 0.013 | -0.67 | 0.92 | 0.75 | 0.04 | -0.77 | 0.85 | 0.93 | 0.03 | -0.76 | 0.83 | 0.93 | |
| **Alcohol consumption** |  |  |  |  |  |  |  |  |  |  |  |  | |
| No | reference |  |  |  | reference |  |  |  | reference |  |  |  | |
| Yes | 0.47 | -0.43 | 1.36 | 0.30 | 0.38 | -0.58 | 1.34 | 0.43 | 0.51 | -0.46 | 1.48 | 0.30 | |
| **Oral contraceptives** |  |  |  |  |  | | | |  |  |  |  | |
| No | reference |  |  |  | n.a. | | | | reference |  |  |  | |
| Yes | -0.71 | -1.64 | 0.23 | 0.14 |  | | | | -0.81 | -1.78 | 0.15 | 0.099 | |

**S3 Table. Stratified analysis for girls (n=408).** HbA1c was included as a continuous variable (mmol/mol) in the regression model. Model 1 was adjusted for age. For birth weight: model 2 was additionally to model 1 adjusted for parental SES. For all variables except birth weight: model 2 was additionally to model 1 adjusted for parental SES, lifestyle factors (smoking, HFD index, sport activity, alcohol consumption) and BMI. Estimates for age and parental SES shown in model 2 are based on the latter comprehensively adjusted model. For all variables except birthweight: model 3 was additionally to model 2 adjusted for oral contraceptive use.
