# Supplementary material for: Distribution and determinants of glycosylated hemoglobin in adolescents ‐ Results from a nationwide population-based survey in Germany
Source: PLoS One. 2024 Feb 22;19(2):e0296962. doi: 10.1371/journal.pone.0296962 (PMC10883580; doi:10.1371/journal.pone.0296962)
Supplement: S4 Table — HbA1c was included as a continuous variable (mmol/mol) in the regression model. Model 1 was adjusted for age. For birth weight: model 2 was additionally to model 1 adjusted for parental SES. For all variables except birth weight: model 2 was additionally to model 1 adjusted for parental SES, lifestyle factors (smoking, HFD index, sport activity, alcohol consumption) and BMI. Estimates for age and parental SES shown in model 2 are based on the latter comprehensively adjusted model. (DOCX) [file pone.0296962.s004.docx]

|  | **Model 1** | | | | **Model 2** | | | |
| --- | --- | --- | --- | --- | --- | --- | --- | --- |
|  | **β** | **95% CI** | | **p-value** | **β** | **95% CI** | | **p-value** |
| **Age (years)** |  |  |  |  |  |  |  |  |
| 14 | reference |  |  |  | reference |  |  |  |
| 15 | 0.03 | -0.98 | 1.03 | 0.96 | 0.16 | -0.95 | 1.27 | 0.78 |
| 16 | -0.67 | -1.81 | 0.47 | 0.25 | -0.29 | -1.50 | 0.92 | 0.64 |
| 17 | -1.25 | -2.54 | 0.03 | 0.057 | -1.04 | -2.40 | 0.32 | 0.13 |
| **Parental socioeconomic status** |  |  |  |  |  |  |  |  |
| Low | reference |  |  |  | reference |  |  |  |
| Medium | 0.73 | -0.76 | 2.22 | 0.33 | 0.88 | -0.55 | 2.30 | 0.23 |
| High | 0.75 | -0.85 | 2.35 | 0.36 | 1.09 | -0.36 | 2.54 | 0.14 |
| **Birth weight** **(g)** |  |  |  |  |  |  |  |  |
| < 2500 | 1.96 | -0.83 | 2.75 | 0.29 | 0.89 | -0.87 | 2.65 | 0.32 |
| 2500 to < 4000 | reference |  |  |  | reference |  |  |  |
| ≥4000 | 1.25 | 0.06 | 2.43 | 0.039 | 1.27 | 0.07 | 2.47 | 0.038 |
| **Body mass index** |  |  |  |  |  |  |  |  |
| BMI**-**SDS | 0.30 | -0.16 | 0.77 | 0.20 | 0.35 | -0.10 | 0.79 | 0.13 |
| **Smoking** |  |  |  |  |  |  |  |  |
| No | reference |  |  |  | reference |  |  |  |
| Yes | 1.66 | 0.50 | 2.81 | 0.005 | 1.48 | 0.36 | 2.61 | 0.010 |
| **Diet** |  |  |  |  |  |  |  |  |
| HFD Index | -2.25 | -5.89 | 1.44 | 0.23 | -2.23 | -5.67 | 1.21 | 0.20 |
| **Sport activity** |  |  |  |  |  |  |  |  |
| No | reference |  |  |  | reference |  |  |  |
| Yes | -0.13 | -1.18 | 0.91 | 0.80 | 0.02 | -1.04 | 1.09 | 0.96 |
| **Alcohol consumption** |  |  |  |  |  |  |  |  |
| No | reference |  |  |  | reference |  |  |  |
| Yes | -0.52 | -1.68 | 0.64 | 0.38 | -0.73 | -1.90 | 0.44 | 0.22 |

**S4 Table. Stratified analysis for boys (n=314).** HbA1c was included as a continuous variable (mmol/mol) in the regression model. Model 1 was adjusted for age. For birth weight: model 2 was additionally to model 1 adjusted for sex, age, and parental SES. For all variables except birth weight: model 2 was additionally to model 1 adjusted for parental SES, lifestyle factors (smoking, HFD index, sport activity, alcohol consumption) and BMI. Estimates for age and parental SES shown in model 2 are based on the latter comprehensively adjusted model.
